# Children’s screen time alters the expression of saliva extracellular miR-222 and miR-146a

**DOI:** 10.1038/s41598-018-26351-2

**Published:** 2018-05-29

**Authors:** Annette Vriens, Eline B. Provost, Nelly D. Saenen, Patrick De Boever, Karen Vrijens, Oliver De Wever, Michelle Plusquin, Tim S. Nawrot

**Affiliations:** 1 Centre for environmental sciences, Hasselt University, Hasselt, Belgium; 2 Environmental risk and health, Flemish Institute for Technological Research, Mol, Belgium; 3 Laboratory of Experimental Cancer Research, Department of Radiation Oncology and Experimental Cancer Research, Ghent University Hospital, Ghent, Belgium; 4 Department of public health and primary care, Leuven University, Leuven, Belgium

## Abstract

An imbalance between energy uptake and energy expenditure is the most important reason for increasing trends in obesity starting from early in life. Extracellular miRNAs are expressed in all bodily fluids and their expression is influenced by a broad range of stimuli. We examined whether screen time, physical activity and BMI are associated with children’s salivary extracellular miR-222 and miR-146a expression. In 80 children the extracellular fraction of saliva was obtained by means of differential centrifugation and ultracentrifugation. Expression levels of miR-222 and miR-146a were profiled by qPCR. We studied the association between children’s salivary extracellular miRNA expression and screen time, physical activity and BMI using mixed models, while accounting for potential confounders. We found that higher screen time was positively associated with salivary extracellular miR-222 and miR-146a levels. On average, one hour more screen time use per week was associated with a 3.44% higher miR-222 (95% CI: 1.34 to 5.58; p = 0.002) and 1.84% higher miR-146a (95% CI: −0.04 to 3.75; p = 0.055) level in saliva. BMI and physical activity of the child were not significantly associated with either miR-222 or miR-146a. A sedentary behaviour, represented by screen time use in children, is associated with discernible changes in salivary expression of miR-146a and or miR-222. These miRNA targets may emerge attractive candidates to explore the role of these exposures in developmental processes of children’s health.

## Introduction

A rising prevalence in obesity, both in children and adults has emerged over the last decade^1^. Obesity fosters the development of a variety of morbidities, among which type 2 diabetes and cardiovascular disease^2^. The onset of these diseases could occur already early in life, with obesity being an important risk factor^3^.

An unhealthy lifestyle, with an imbalance between energy intake and expenditure is the predominant cause of obesity. Insufficient energy expenditure can directly be linked to a lack of physical activity^4^. Although it is well-accepted physical activity is an effective measure to reduce multiple health risk factors, a sedentary lifestyle is frequent in all age groups^5^. Children spend a substantial amount of time in sedentary pursuits, such as watching television, using the computer and playing videogames. This behaviour not only increases the risk to develop obesity, but also increases the risk to develop metabolic syndrome and cardiovascular disease^6^.

miRNAs are short (~22 nt long) single-stranded RNA molecules which control gene networks and play a central role in a various (patho)physiological processes^7^. miRNAs are present both intracellular and in the extracellular environment. Extracellular miRNAs, which are protected from degradation and carried by vesicles and protein aggregates, are stably present in all bodily fluids. Recently, they are recognized as important signal molecules and/or biomarkers in the pathology of various morbidities^8^. As such, circulating miRNAs are implemented in cardiometabolic disease mechanisms^9^. Studies focusing on differential miRNA expression patterns in patients with various cardiometabolic disorders compared with healthy subjects showed the importance of miR-222 and miR-146a. In this regard, the inflammatory miR-146a is linked to metabolic and cardiovascular disease^9^. miR-222 is a cell cycle regulator, which is involved in both physiological and pathological cardiac processes^10^. The (extracellular) expression profiles of miRNAs in body fluids are suggested as potential biomarkers for cardiometabolic processes. However, these studies either focus on subjects already having a specific metabolic or cardiovascular disorder or on specific interventions in adults. Studies linking miR-146a or miR-222 with lifestyle factors related to cardiometabolic outcomes in childhood are currently lacking. We investigated whether miR-146a and miR-222 in the extracellular fraction of saliva are associated with screen time use, physical activity and BMI in children.

## Methods

### Study population

This study was based on the COGNAC (COGNition and Air pollution in Children) study^11–13^, which is a panel study with three repeated measures. Between 2011 and 2013, we invited children (grades three to six) from three primary schools in Flanders (Belgium) to participate. 43.4% of all invited children participated in the study. The examinations took place between December 2011 and February 2014 on Monday, Tuesday, Thursday, and Friday between 9:00 a.m. and 2:00 p.m. Of the 334 children within the COGNAC cohort, we randomly selected 80 children, from two participating schools. Saliva samples of the first two study visits, which were approximately 3 months apart, were selected. For each child, all visits were scheduled at the same day of the week and same time point, to rule out diurnal variation. The study was approved by the medical ethical committee of Hasselt University and the Eastern-Limburg Hospital (Belgium) in accordance with the Helsinki Declaration^14^. Parental informed consent was obtained prior to participation in the study. Information on the socio-economic status (by parental education and occupation), exposure to second-hand smoke through parental smoking and the use of TV and PC screens was accessible by a questionnaire filled out by the parents. Based on the reported weekly TV and PC screen use, we calculated the total screen time as the sum of TV and PC screen use. Based on information of the reported sport activities, out-of-school sport activities were identified and categorized as “none” (i.e. no out-of-school sport activities), “low” (i.e. ≤3 hours per week), “middle” (>3 to <6 hours per week) and high (≥6 hours per week). Passive smoking was defined as exposure to indoor tobacco smoke, when one or more family member(s) smoked inside the house. Height and weight were recorded and body mass index (BMI) was calculated. Overweight and obesity were defined according to international childhood BMI thresholds^15^.

### Saliva extracellular miRNAs

In order to avoid contamination of the samples, children refrained at least 30 minutes from eating, drinking or hygienic procedures prior to saliva donation. Additionally, they rinsed three times with tap water to eliminate possible food residues. Saliva (2 ml) was collected using the Oragene® RNA self-collecting kits (DNA Genotek Inc.) and immediately stabilized by mixing with RNA stabilizer. Within 6 hours, the samples were stored at −20 °C until further analyses.

The extracellular fraction of the saliva was obtained by differential centrifugation and ultracentrifugation, protocol was adapted from Théry *et al*.^16^ to integrate the processing of saliva specific to the Oragene collection kits in the procedure. After thawing, the saliva was incubated at 50 °C for one hour. Subsequently, a 1 ml aliquot was incubated at 90 °C for 15 minutes. Next, the debris present in saliva was pelleted by adding 40 µl of neutralizer solution (DNA Genotek Inc.) and centrifuging samples at 1500 × *g* for 10 minutes. The supernatant was collected and centrifuged at 16000 × *g* for 20 minutes. Then, the supernatant was ultracentrifuged at 160000 × *g* for one hour (Optima LE-80 K ultracentrifuge equipped with a ti70 fixed angle rotor; Beckman). The polyallomer tubes for ultracentrifugation were pre-treated with RNA*Zap* (Life Technologies) to remove RNAse activity. After the first ultracentrifugation step, the pellet was resuspended in 1x PBS (pH 7.4) and ultracentrifuged at 160000 × *g* for one hour. Afterwards, the pellet, containing vesicles and protein aggregates^17^, was resuspended in RNAse-free water and stored at −80 °C.

Total RNA, including small miRNAs was isolated from the extracellular fraction of saliva using the miRNeasy mini kit (Qiagen), following the manufacturer’s instructions. Samples were spiked with 250 fmol *C. elegans miR-39* for normalization of the expression data. 125 ng total RNA was reverse transcribed using looped primers for specific miRNA cDNA synthesis (Megaplex RT primers human pool A & Taqman mircoRNA RT kit; Life Technologies) on a PCR gradient thermal cycler (TC-5000; Techne). For all samples, 1:75 dilutions were made and an equal volume of products was mixed with reagents of the Taqman miRNA assay and the Taqman Fast Advanced mastermix (Life Technologies) for quantification of the miRNAs. qPCR was carried out on an ABI 7900HT sequence detection system (Life Technologies) and thermal cycling was for 10 minutes at 95 °C, followed by 40 cycles of 15 seconds at 95 °C and one minute at 60 °C. All runs were carried out in triplicate and with a no-template control (NTC) on 384-well plates with three inter run calibrators (IRC). Raw qPCR data were analysed using the SDS Relative Quantification Software (version 2.3; Applied Biosystems). Cq values were transformed to a relative quantity to the external spike-in miRNA *cel-miR-39* in qbase+ software (Biogazelle).

### Statistical analysis

We used SAS (version 9.3, SAS institute Inc., Cary, NC, USA) software for data management and statistical analyses. Demographic characteristics are represented as mean (standard deviation) for continuous variables or number (frequency) for categorical variables. miRNA expression data were log_10_-transformed to improve normality of the data.

**Figure 1:**
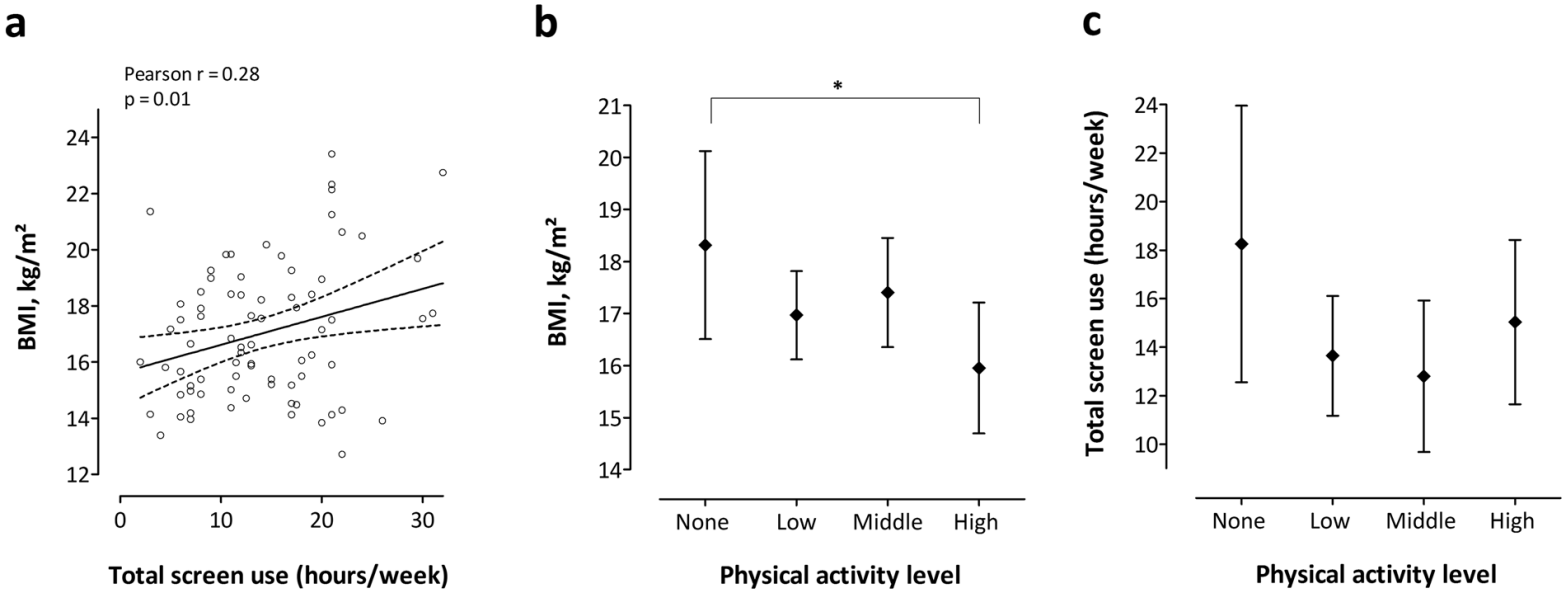
Correlation between total screen use, physical activity and body mass index (BMI). (**a**) Correlation between total screen use and BMI (line represents the linear fit of the correlation with the 95% confidence intervals), (**b**) Mean (95%CI) BMI in function of the physical activity level category, adjusted for age and gender of the child, (**c**) Mean (95%CI) total screen time in function of the physical activity level. *p < 0.05.

We correlated the total screen time use with BMI (Fig. 1a) and we evaluated the associations of screen time, physical activity and the BMI of the child using linear regression models, taking into account children’s age and gender. Using the LSMEANS option, we studied mean BMI values (with 95% confidence intervals (CI), Fig. 1b) while accounting for gender and age and mean screen time values (with 95% CI, Fig. 1c) for each physical activity category.

We evaluated the possible associations between screen time, BMI and physical activity and salivary extracellular miR-222 and miR-146a expression using the MIXED procedure to account for the hierarchical structure of the data (i.e. repeated measures for the miRNAs). This implies correlation between measurements within the same child, but no correlation between different children. The child identifier was included as a random effect nested within the schools in the mixed model. Restricted maximum likelihood estimation (REML) with unstructured autocorrelation was employed to estimate the coefficients and standard errors. A priori selected covariates were added to the model as fixed effects to correct for possible confounding. In models evaluating the associations between screen use/BMI/physical activity and miRNA expression as a dependent variable, the following variables were chosen: age of the child (continuous), gender, maternal education (two categories: up to high school diploma/college or university diploma), passive smoking (yes/no) and the extracellular RNA concentration. Effect estimates of significant covariates were presented based on the model that evaluated the effect of screen time. The effect estimates are represented as a percentage change in extracellular miRNA expression. Q-Q plots of the residuals were checked to test the assumptions of the models. Due to variability and heterogeneity in gene expression profiles, it is difficult to distinguish true gene expression alterations associated with the outcome from passenger signals^18^. To study the robustness, we applied Monte Carlo simulation (10,000 times) for adjustment of the parameter estimates of the significant predictors of miRNA expression using the PLM procedure. Finally, we evaluated the prediction of the models using receiver-operating characteristics (ROC) plots. The children were stratified according to their reported screen time use with the 75^th^ percentile as a cut-off point (19.5 hours per week). The ROC plots of the models are provided (Supplementary information).

## Results

The characteristics of the study population are reported in Table 1. The number of boys and girls included in the study were approximately equal. The average (standard deviation) age of the children was 10 (1) years and BMI averaged 17 (2.4) kg/m². Of the 80 children, 14 (17.5%) were underweight and 10 (12.5%) were overweight. 9 children were exposed to tobacco smoke by parental indoor smoking. The average TV screen use was 9.3 (5.5) hours per week, and average PC screen use was 4.8 (3.7) hours per week. The TV screen use was not correlated with the PC screen use (Pearson correlation coefficient r = 0.11; p = 0.34). Total screen use, defined by the sum of TV and PC use, is on average 14.11 (6.93) hours per week.

**Table 1:** Demographic characteristics of the study population (n = 80).

	Mean (SD) or n (%)
Boys	37 (46.3%)
Age, yrs	10.44 (0.97)
BMI, kg/m²^ a^	17.01 (2.42)
Underweight	14 (17.5%)
Normal weight	56 (70%)
Overweight	10 (12.5%)
Passive smoking	9 (11.3%)
Maternal level of education	
Up to high school diploma	26 (32.5%)
College or university diploma	54 (67.5%)
Caucasian	73 (91.3%)
TV screen use, hours per week	9.32 (5.46)
Computer screen use, hours per week^b^	4.77 (3.65)
Total screen use, hours per week^b^	14.11 (6.93)
Physical activity^c^	
None	7 (9%)
Low	33 (42.3%)
Middle	21 (26.9%)
High	17 (21.8%)

^a^BMI categorization based on age and gender specific children’s growth curves for Flanders 2004; ^b^n = 77 (included in the analysis); ^c^low: ≤3 hours, middle: >3 and <6 hours per week, high: ≥6 hours per week, n = 78.

Both before (Fig. 1a) and after adjustment of BMI for child’s age and gender, total screen use was positively correlated with children’s BMI. Each one hour increment of screen use per week was associated with a 0.08 kg/m² higher BMI (95% CI: 0.004–0.163; p = 0.04), independent of children’s age and gender. Compared with children without out-of-school physical activities, children with a high activity level have a 2.37 kg/m² lower BMI (95% CI: −4.56–0.17; p = 0.0.4, overall p = 0.16) (Fig. 1b). Physical activity levels were not associated with the total screen time use (overall p = 0.36; Fig. 1c).

The association between total screen time use, BMI and physical activity on salivary extracellular miR-146a and miR-222 expression was evaluated using a mixed model, which took into account the repeated measurements of the miRNAs for each child, while accounting for gender, age, passive smoking, maternal education and the RNA content of saliva (Table 2).

**Table 2:** The estimated changes in miR-222 and miR-46a associated with screen time, BMI and physical activity as evaluated in separate models.

	Effect size (95% CI)^*^	p-value
**miR-222**		
Total screen time	3.44 (1.34–5.58)	0.0016
BMI, continuous	−2.20 (−8.07–4.04)	0.48
BMI, categorical		0.59
Underweight	18.51 (−18.57–72.46)	
Normal weight	Ref	
Overweight	13.26 (−25.97–73.30)	
Physical activity		0.74
None	Ref	
Low	10.19 (−35.15–87.24)	
Middle	−2.74 (−44.31–69.82)	
High	−12.75 (−51.85–58.09)	
**miR-146a**		
Total screen time	1.84 (−0.04–3.75)	0.055
BMI, continuous	−4.08 (−8.75–0.84)	0.10
BMI, categorical		0.43
Underweight	12.74 (−16.88–52.90)	
Normal weight	Ref	
Overweight	−15.35 (−40.23–19.90)	
Physical activity		0.71
None	Ref	
Low	27.67 (−17.99–98.79)	
Middle	15.97 (−28.12–87.07)	
High	19.81 (−27.22–97.24)	

^*^Effect sizes represent the mean% change with the 95% confidence intervals in miRNA for a 1-hour increment in weekly screen time/1 kg/m² increase in BMI or compared to a reference group. Models were adjusted for gender, age, passive smoking exposure, maternal education and RNA content of the extracellular fraction.

Total weekly screen time use was significantly associated with salivary extracellular miR-146a and miR-222 expression. One hour increase in weekly screen time was associated with 3.44% higher (95% CI: 1.34–5.58; p = 0.002) extracellular miR-222 levels in saliva. One hour increase in weekly screen time was associated with 1.84% higher (95% CI: −0.04–3.75; p = 0.055) levels of salivary extracellular miR-146a. Monte Carlo adjusted confidence intervals and p-values for the associations of the miRNAs with screen time are confirmative and given in Table 3. No significant association between either BMI or physical activity and miR-222 and miR-146a was observed.

**Table 3:** The estimated changes in miR-222 and miR-146a associated with screen time, adjusted using Monte Carlo simulation.

	Effect size (adjusted 95% CI)	Adjusted p-value
miR-222	3.44 (1.34–5.62)	0.0022
miR-146a	1.84 (−0.07–3.78)	0.060

^*^Effect sizes represent the mean % change with the 95% confidence intervals in miRNA for a 1-hour increment in weekly screen time. Models were adjusted for gender, age, passive smoking exposure, maternal education and RNA content of the extracellular fraction. Monte Carlo simulation was repeated 10,000 times.

In addition to screen time use, salivary extracellular miR-222 was significantly higher in children exposed to passive smoking. On average, passive smoking was associated with 55% higher (95% CI: 1–139; p = 0.044) miR-222 levels in the extracellular fraction of the saliva. Age of the child was inversely associated with both miR-222 and miR-146a. A 1 year increase in age was associated with a 16.11% lower (95% CI: −27.15 to −3.41; p = 0.01) saliva extracellular level of miR-222 and a 15.86% lower (95% CI: −25.95 to −4.37; p = 0.01) saliva extracellular level of miR-146a.

## Discussion

Already from childhood onwards, sedentary behaviour is associated with adverse health outcomes^19^. From both intervention and longitudinal studies it is apparent that watching TV is related to overweight and obesity in children^19^. A meta-analysis including 170 studies in children, indicated a reduction in BMI with a reduction in the sedentary behaviour^19^. Due to their established role in developmental processes miRNAs have emerged as attractive candidates to explore the impact of exposures during critical windows of susceptibility^7^. 

In this context the key finding of our current paper is that children’s weekly screen time use is positively associated with BMI and this parallels differential profiles of salivary extracellular miR-146a and miR-222 expression.

The relevance of these miRNAs in the framework of physical (in)activity becomes clear from studies in adults on acute exercise^20–22^ and fitness^23^, obesity^24^, atherosclerosis^25^, and diabetes^26–30^. For example, after acute exercise a transient up-regulation in circulating levels of miR-146a and miR-222 is observed^20,22^. Furthermore, this upregulation is also observed short-term after a training session in athletes on a sustained training schedule^20^. miR-222 can be indicative of a subject’s fitness level based on its positive association with the maximal oxygen uptake (VO_2max_) in healthy adults^23^. Compared to normal weight subjects, circulating miR-146a^31^ and miR-222 levels^24^ can be alternatively expressed in obese subjects. Many studies report altered expression levels of miR-146a in type 2 diabetes patients^26–30,32^. The functional significance of changes in the expression of these miRNAs in the extracellular fraction of saliva remains to be elucidated. Extracellular miRNAs could be involved in cell communication, but the process is currently poorly understood. Extracellular miRNAs are promoted as promising biomarkers^8^, also for aspects related to lifestyle and diet^33^. Alterations in the (extracellular) miRNA abundance can reflect a dynamic response to metabolic stress in an attempt to re-establish homeostasis or a continuing response involved in disease development.

The findings of this study should be interpreted in the context of its limitations and strengths. As with other studies investigating the effect of screen time use or physical activity, the use of self-reported quantities of screen time and sport activities might give some exposure misclassification. In our study, physical activity was based on the reported out-of-school sport activities. It is important to note, that apart from the out-of-school sport activities other leisure activities contribute to the total physical activity level of the child. Future studies could benefit from the implementation of activity trackers and diaries, to measure physical inactivity in a more objective manner. Second, we only quantified two miRNAs, which is insufficient to draw conclusions on biological mechanisms contributing to the early onset of adult disease. Finally, observational studies cannot prove the utility of a biomarker. Nevertheless, our proof-of-concept findings indicate changes in extracellular miRNAs in relation to childhood life-style factors that are particularly relevant in the development of obesity. miR-222 and miR-146a have been implicated in cardiovascular health outcomes in adults. Our findings add to this by highlighting a correlation between these miRNAs and lifestyle in healthy children, suggesting that their expression profiles might be linked to cardiometabolic processes, already from early-life onwards. Therefore there is a possible role of these miRNAs in translating the impact of sedentary behaviour on our genome, which should be further validated in intervention studies. Gene expression can be highly heterogeneous and this hampers capturing true alterations in gene expression associated with health outcomes or lifestyle factors instead of passenger signals reflecting the heterogeneity^18^. We confirmed the robustness of our findings by employing a statistical resampling technique. A strength of our study is that we explored a non-invasive matrix by use of saliva which is preferable in population based studies including children.

## Conclusion

To conclude, we showed expression of two extracellular miRNAs in saliva is associated with screen time use in children. The alterations in the salivary miRNA signature reflects epigenetic alterations related to an increased sedentary behaviour in school-aged children, which is particularly relevant since these miRNAs are associated with fitness and metabolic health in adults. Further research on this topic is warranted to understand the mechanism underlying the effects early-life inactivity on epigenetic alterations.

## Electronic supplementary material

Supplemental information
