# Supplementary material for: Children’s screen time alters the expression of saliva extracellular miR-222 and miR-146a
Source: Sci Rep. 2018 May 29;8:8209. doi: 10.1038/s41598-018-26351-2 (PMC5974392; doi:10.1038/s41598-018-26351-2)
Supplement: Supplementary file 1 — Supplemental information [file 41598_2018_26351_MOESM1_ESM.docx]

Children’s screen time alters the expression of saliva extracellular miR-222 and miR-146a

Annette Vriens, Eline B. Provost, Nelly D. Saenen, Patrick De Boever, Karen Vrijens, Oliver De Wever, Michelle Plusquin, Tim S. Nawrot

**SUPPLEMENTAL INFORMATION**

**CONTENT:**

Supplemental table 1: Logistic regression between high and low screen time use in association with saliva miR-146a and miR-222

Supplemental figure 1: Receiver Operating Characteristic (ROC) curves to indicate the predictive power of our model to differentiate between individuals with high (>75th percentile) and low (<75th percentile) reported screen time use. (A) Performance of the model including all covariates (age, gender, maternal education, passive smoking exposure and extracellular RNA content). (B) Performance of the model including miR-222 and covariates. (C) Performance of the model including miR-146a and covariates. (D) Performance of the model including both miR-146a and miR-222 and the covariates.

***Supplemental table 1: Logistic regression between high and low screen time use in association with saliva miR-146a and miR-222***

|  | Odds ratio (95% CI) ^c^ | p-value |
| --- | --- | --- |
| miR-222 ^a^ | 6.39 (1.58 – 25.82) | 0.0092 |
| miR-146a ^b^ | 2.70 (0.37 – 3.61) | 0.33 |

*Screen time use was dichotomized into two groups: high (>P75 reported screen time use) and low (<P75 reported screen time use). The logistic regression model was adjusted for age and gender, maternal education, exposures to passive smoking and the extracellular RNA content. ^a^: the results correspond to panel C of the supplemental figure 1; ^b^: the results correspond to panel B of the supplemental figure 1; ^c^: the odds ratios for having a high screen time use are expressed for a 10-fold increment in microRNA expression.*

***
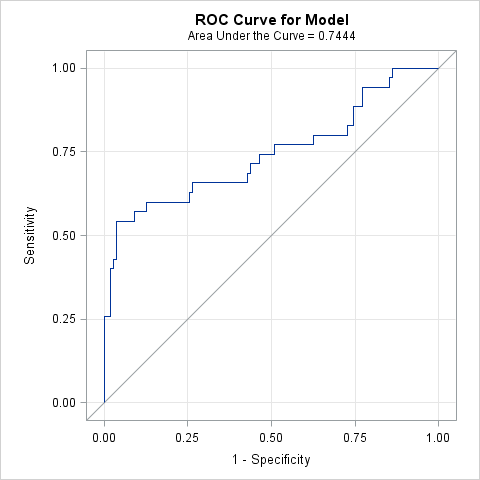

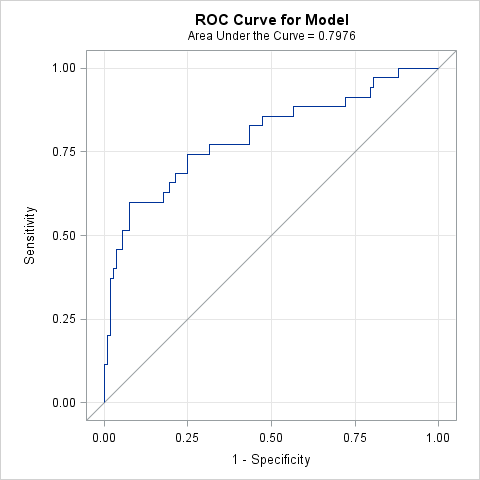
***

**B**

**A**

***
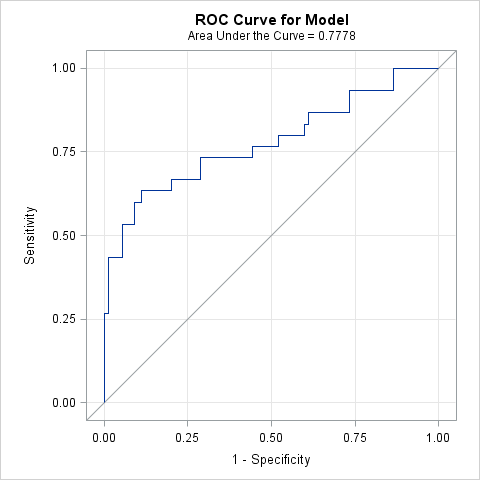

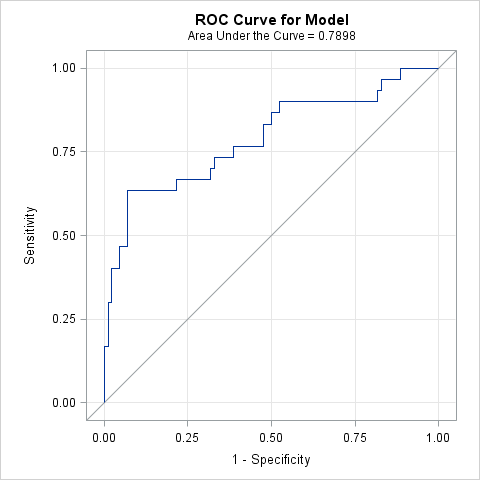
***

**C**

**D**

***Supplemental figure 1: Receiver Operating Characteristic (ROC) curves to indicate the predictive power of our model to differentiate between individuals with high (>75th percentile) and low (<75th percentile) reported screen time use.*** *(A) Performance of the model including all covariates (age, gender, maternal education, passive smoking exposure and extracellular RNA content). (B) Performance of the model including miR-222 and covariates. (C) Performance of the model including miR-146a and covariates. (D) Performance of the model including both miR-146a and miR-222 and the covariates.*
